# New Eco-Friendly Phosphorus Organic Polymers as Gas Storage Media

**DOI:** 10.3390/polym9080336

**Published:** 2017-08-03

**Authors:** Dina S. Ahmed, Gamal A. El-Hiti, Emad Yousif, Ayad S. Hameed, Mustafa Abdalla

**Affiliations:** 1 Department of Chemistry, College of Science, Tikrit University, Tikrit, Iraq; dinasaadi86@gmail.com (D.S.A.); ch@sc.nahrainuniv.edu.iq (A.S.H.); 2 Cornea Research Chair, Department of Optometry, College of Applied Medical Sciences, King Saud University, P.O. Box 10219, Riyadh 11433, Saudi Arabia; 3 Department of Chemistry, College of Science, Al-Nahrain University, Baghdad 64021, Iraq; msar83@gmail.com

**Keywords:** eco-friendly polymers, phosphorus polymers, gas storage, gas uptake, gas capture, Brunauer–Emmett–Teller surface area

## Abstract

Three phosphate esters **1**–**3** were successfully synthesized from the reaction of 2-, 3- and 4-hydroxybenzaldehyde with phosphoryl chloride. Reactions of **1**–**3** with benzidine in the presence of glacial acetic acid gave the corresponding novel phosphorus organic polymers **4**–**6** containing the azomethane linkage. The structures of the synthesized compounds were confirmed by Fourier transform infrared spectroscopy, nuclear magnetic resonance and elemental analysis. Interesting physiochemical properties for the polymeric materials **4**–**6** were observed using a combination of several techniques such as gel permeation chromatography, scanning electron microscopy, Brunauer–Emmett–Teller and nitrogen adsorption–desorption isotherm, Barrett–Joyner–Halenda and H-sorb 2600 analyzer. The mesoporous polymers **4**–**6** exhibit tunable porosity with Brunauer–Emmett–Teller surface area (SA_BET_ = 24.8–30 m^2^·g^–1^), pore volume (0.03–0.05 cm^3^·g^–1^) and narrow pore size distribution, in which the average pore size was 2.4–2.8 nm. Polymers **4**–**6** were found to have high gas storage capacity and physico-chemical stability, particularly at a high pressure. At 323 K and 50 bars, polymers **4**–**6** have remarkable carbon dioxide uptake (up to 82.1 cm^3^·g^–1^) and a low hydrogen uptake (up to 7.4 cm^3^·g^–1^). The adsorption capacity of gasses for polymer **5** was found to be higher than those for polymers **4** and **6**.

## 1. Introduction

Carbon dioxide (CO_2_) emission levels in the atmosphere are increasing mainly as a result of high fossil fuel consumption. Carbon dioxide is considered one of the main greenhouse gases (GHG) which causes global warming [1,2,3,4,5,6,7,8]. This has led to climate changes such as flooding and droughts due to the changes in nature’s equilibrium. Recently, research has been directed to find ways to capture GHGs such as CO_2_, which can reduce the efficiency and capacity of natural gas and lead to gas piping corrosion [9,10]. Various strategies have been developed to reduce CO_2_ emission, one of the most common methods for capturing CO_2_ followed by underground storage is known as carbon capture and storage (CCS) [11]. Therefore, the production of new materials that are not only capable of capturing CO_2_ but also act as storage for clean energy is always of interest. The most common gas mixtures that require separation and capture technology are: natural gas, which is composed of methane (80–95%) along with CO_2_ as the major impurity; flue gas, which contains nitrogen (70%) along with CO_2_ (10–15%); and pre-combustion gas mixture, which contains hydrogen (H_2_) [12,13,14,15,16].

Hydrogen is considered one of the cleanest sources of energy in which water is the only product arising from its combustion [17]. Therefore, the development of efficient and safe methods for hydrogen storage is still important [18]. One conventional approach to hydrogen gas storage involves chemisorption of hydrogen, for which metal chemical hydrides have been investigated [19]. An alternative approach involves physisorption of hydrogen in porous media such as porous carbons, which have high stability and are easily accessible [20,21]. Recently, many efforts have been made to develop porous organic polymers (POPs) for their use in gas storage and separation, heterogeneous catalysis, light-harvesting, molecular sensors and other interesting applications [22,23,24,25,26,27,28,29,30,31]. Two main strategies are used in the design and production of POP materials. The first one involves the selection of suitable monomers and the second involves the choice of proper synthesis method that is highly flexible [22]. The main goal of using these efficient strategies is to control the porosity and functionality of the synthesized polymeric materials. Monomers should have multifunctional reaction sites with rigid or contorted structures [22]. The monomers that can afford high porosity could have linear [32], planar [33], tetrahedral [34] or octahedral geometries [35]. Metal–organic framework (MOF) compounds contain organic ligands that coordinate to metal ions and have interesting properties: they have small and tunable pore sizes, and can be used in various applications such as an efficient absorbent to capture H_2_ and CO_2_ [36,37,38,39].

Phosphorus–organic polymers have good mechanical and fire resistance properties [40]. They can be used as fire retardants, flame proofers, surface adhesion reagents, catalysts and tooth preservers [40,41,42,43,44,45]. To the best of our knowledge, no attempts have been made to use phosphorus–organic polymers for gas storage. Therefore, as a continuation of our research in the use of polymeric materials in various applications [46,47,48,49,50], we were interested in designing and synthesizing new polymeric materials containing phosphorous to be used as efficient gas storage. The current study manages CO_2_ capture to reduce greenhouse gas emission, which is an environmental issue of a major concern.

## 2. Experimental Section

### 2.1. Instrumentation

The FT-IR spectra were recorded using Shimadzu 8400 spectrophotometer (400–4000 cm^–1^) using KBr disks technique. Proton nuclear magnetic resonance (^1^H NMR; 400 MHz) spectra were recorded on a Bruker DRX400 NMR spectrometer (Zürich, Switzerland) in DMSO-*d_6_*. Elemental analyses were performed using Vario EL III Elementar instrument. Scanning electron microscopy (SEM) observations were carried out using a KYKY-EM3200 microscope (Ontario, CA, USA) at an accelerating voltage of 26 kV. The molecular weight was determined by gel permeation chromatography (GPC) using a Tosoh Bioscience GmbH EcoSEC HLC-8320GPC system (Tokyo, Japan). The system was equipped with a refractive index (RI) and an ultraviolet (UV) detector (λ = 280 nm) using a Tosoh TSK gel Alpha-4000 and 2000 columns (300 mm × 7.8 mm ID, Griesheim, Germany), with a particle size of 10 µm and a pore size of 450 Å that operated at 40 °C. Dimethylformamide (DMF) containing LiBr (10 mM) was used as the solvent at a flow rate of 1 mL/min. Calibration curves were obtained using polystyrene standards (*M*n 2.89–500 × 10 ^6^ g/mol).

Nitrogen (N_2_) adsorption–desorption isotherms were recorded at 77 K using Quantchrome analyzer. The samples were dried at 200 °C under dry nitrogen flow for 5 h before measurement. The specific surface areas were calculated following the multipoint Brunauer–Emmett–Teller (BET) method. The pore volumes were determined at a relative pressure (*P/P*_0_) of 0.98. The pore size distributions were verified by a Barrett–Joyner–Halenda (BJH) method. Gas uptake at 323 K and 50 bars were performed by H-sorb 2600 high pressure volumetric adsorption analyzer (Gold APP Instrument Corporation, Beijing, China). The gas adsorption analyzer has two degassing and analyzing ports that work simultaneously. A known amount of gas was dosed into the measurement tube containing the sample. When equilibrium between the sample and adsorbed gas was attained, a comprehensive final equilibrium pressure was automatically recorded using specialist software. The quantity of gas adsorbed was calculated from the data generated. The samples were degassed at 200 °C under dynamic vacuum for 5 h prior to the adsorption tests.

### 2.2. Synthesis of Phosphate Esters ***1***–***3***

To a stirred mixture of phosphoryl chloride (1.53 g, 10 mmol) and triethylamine (3.04 g, 30 mmol) in dry tetrahydrofuran (THF; 15 mL), in a dried three-necked round bottom flask (200 mL) fitted with a thermometer probe and mechanical stirrer, a solution of the appropriate hydroxybenzaldehyde (3.66 g, 30 mmol) in dry THF (15 mL) was added dropwise through a dropping funnel over a period of 20 min at 0 °C. The mixture was stirred at 40–45 °C for 5 h and then left to cool down to room temperature. The filtrate was collected by filtration and the solid, triethylamine hydrochloride, was washed with THF (3 × 25 mL). The combined THF was removed under reduced pressure to give the phosphate esters **1**–**3** as brownish oils in 71–77% yields.

### 2.3. Synthesis of Polymeric Schiff Bases ***4***–***6***

A mixture of phosphate esters **1**–**3** (8.21 g, 20 mmol) and benzidine (5.53 g, 30 mmol) in chloroform (25 mL) containing glacial acetic acid (0.5 mL) was stirred under reflux for 6 h. The mixture was left to cool to room temperature and the solid obtained was filtered, washed with chloroform (3 × 10 mL) and dried to give polymeric materials **4**–**6** as orange powders in 80–86% yields.

## 3. Results and Discussion

### 3.1. Synthesis of Phosphate Polymers ***4***–***6***

**Scheme 1:**
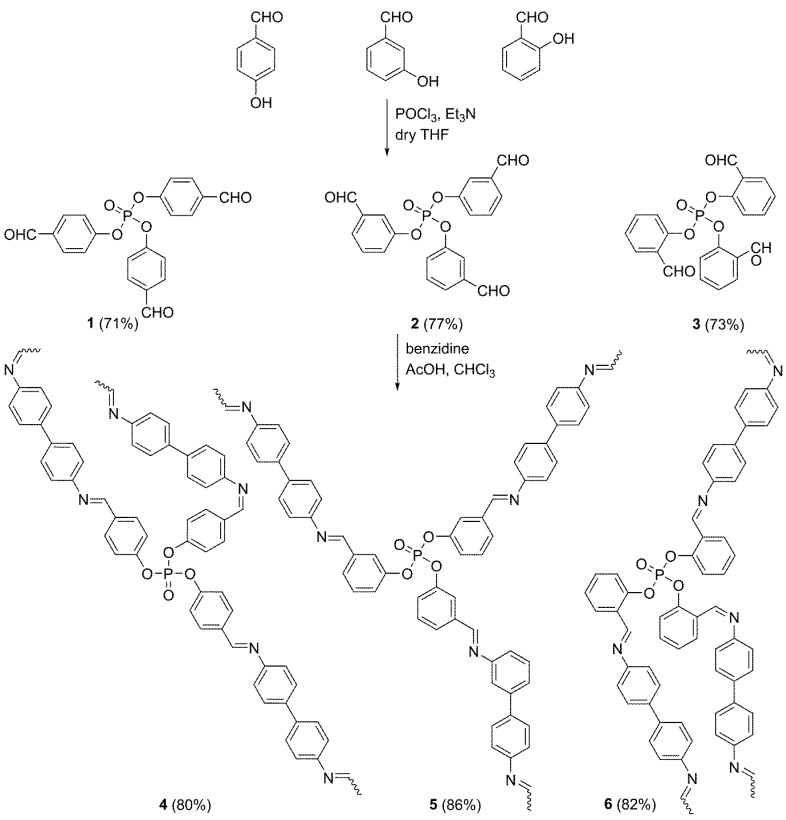
Synthesis of phosphate polymers **4**–**6**.

Three phosphate esters, *tris*(2-formylphenyl)phosphate (**1**), *tris*(3-formylphenyl)phosphate (**2**) and *tris*(4-formylphenyl)phosphate (**3**), were synthesized at 71–77% yields from reactions of 4-, 3- and 2-hydroxybenzaldehyde and phosphoryl chloride, respectively, in dry THF under reflux for 5 h (Scheme 1). Reactions of **1**–**3** with benzidine in chloroform under reflux condition for 6 h gave the corresponding phosphate polymeric Schiff bases **4**–**6** in 80–86% yields. Such phosphate esters can be easily scaled up for various applications and provide three reaction sites, enabling the formation of a crosslinking polymer network.

### 3.2. FT-IR Spectroscopy of Phosphate Esters ***1***–***3***

The FT-IR spectra of phosphate esters **1**–**3** show no hydroxyl groups absorption. The spectra show the presence of carbonyl groups (1681–1693 cm^–1^) and the P–O–C groups (1161–1188 cm^–1^) which is a clear indication that the esterification has taken place. The most common IR absorption bands for phosphate esters **1**–**3** are represented in Table 1 along with their elemental analyses.

### 3.3. ^1^H NMR Spectroscopy of Phosphate Esters ***1***–***3***

The structures of phosphate esters **1**–**3** were also confirmed by the ^1^H NMR spectral data (Table 2). They show singlet signals that resonate at 9.79–10.30 ppm region corresponding to the aldehyde proton. They also show the expected types of aromatic protons.

### 3.4. FT-IR Spectroscopy of Phosphate Polymers ***4***–***6***

The FT-IR spectra of **4**–**6** show the absence of the carbonyl groups which is a clear indication that the polymerization process of the phosphate esters **1**–**3** has taken place. The FT-IR spectra of **4**–**6** show the presence of absorption bands that appear at 1616–1635 cm^–1^ and can be attributed to the azomethane bands (C=N). Some of the most common and intense absorption bands in the FT-IR spectra for **4**–**6** are shown in Table 3.

### 3.5. Molecular Weight and Molecular Distribution of Phosphate Polymers ***4***–***6*** Determined by Gel Permeation Chromatography (GPC)

**Figure 1:**
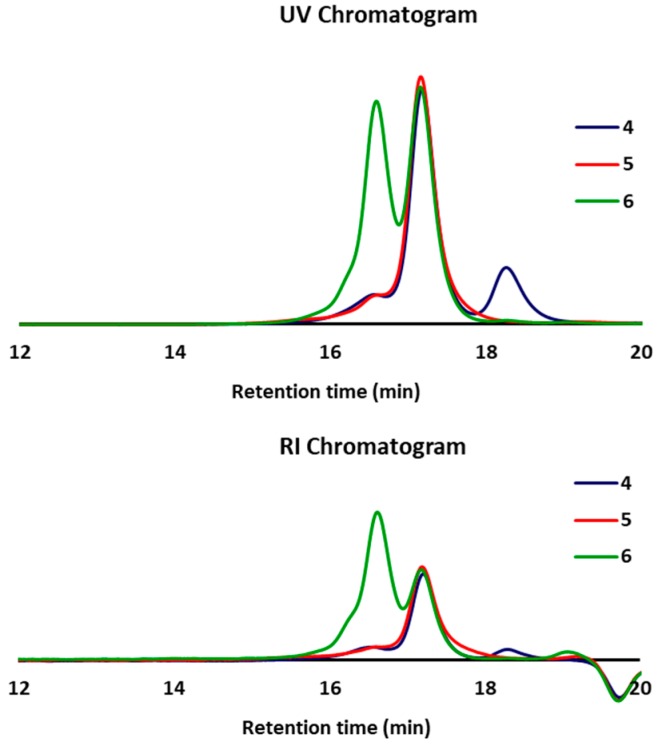
GPC chromatograms of polymers **4**–**6**.

Gel Permeation Chromatography is a good technique to evaluate the molecular weight distribution (MWD) of resins which plays a major role in characterizing the resins performance capacities such as cure speed, viscosity, green strength development, substrates penetration and adhesion [51]. Higher molecular oligomers usually have smaller pores in packing column and can be eluted faster with a small retention time. On the other hand, smaller molecular weight oligomers remain in the pores and eluted slowly with a longer retention time [52]. The GPC chromatograms of polymers **4**–**6** are represented in Figure 1. The number average molecular weight (*Mn*), weight average molecular weight (*Mw*) and polydispersity index (*Dp*) were calculated using Equations (1)–(3) [53] and represented in Table 4.

(1) $$Mn=\frac{\Sigma ni\;Mi}{\Sigma ni}=\frac{\sum_{i=1}^{N}Ai}{\sum_{i=1}^{N}Ai/Mi}=\frac{\sum hi}{\sum \;hi/Mi}$$

(2) $$Mw=\frac{\Sigma ni\;Mi^{2}}{\Sigma ni\;Mi}=\frac{\sum_{i=1}^{N}Ai,Mi}{\sum_{i=1}^{N}Ai}=\frac{\sum_{i=1}^{N}hi,Mi}{\sum_{i=1}^{N}hi}$$

(3) $$Dp=\frac{Mw}{Mn}$$

where *ni* is the number of molecules, *Mi* is the molecular weight, *Ai* is the area of slice *i* in which total area is *A* and *hi* is the peak height at each interval of molecular weight.

### 3.6. Scanning Electron Microscopy (SEM) of Phosphate Polymers ***4***–***6***

**Figure 2:**
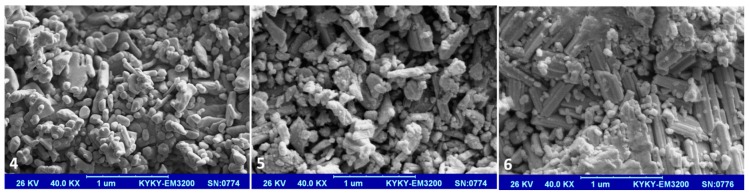
The micrographs of phosphate polymers **4**–**6** (1 µm width).

The morphologies, sizes of particles and porosity of phosphate polymers **4**–**6** were observed by SEM analysis. Higher magnification SEM images revealed the irregular morphology of **4**–**6** (Figure 2) with a grain size that ranged from ten to hundreds of nanometers. The polymers were amorphous and mostly micro-sized particles in which their dimensions ranged 40.0–164.8, 28.4–805.9 and 29.8–598 nm for **4**, **5** and **6**, respectively. The SEM images demonstrate that the polymers consist of agglomerated particles arranged side by side which formed a topographical microporous structures, each particle gathered to form a clusters due to the high surface energy. The SEM images of polymers **4**–**6** exhibit a porous structure due to the azomethane C=N linked network (Figure 2).

### 3.7. Pure Gas Adsorption of Phosphate Polymers ***4***–***6***

**Figure 3:**
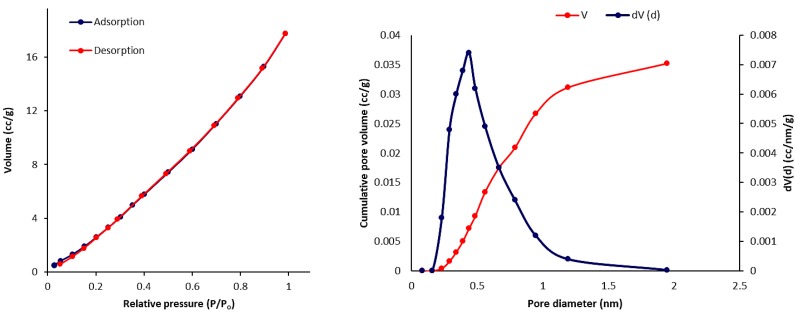
Adsorption–desorption isotherms of N_2_ and pore size distribution curve for **4**.

**Figure 4:**
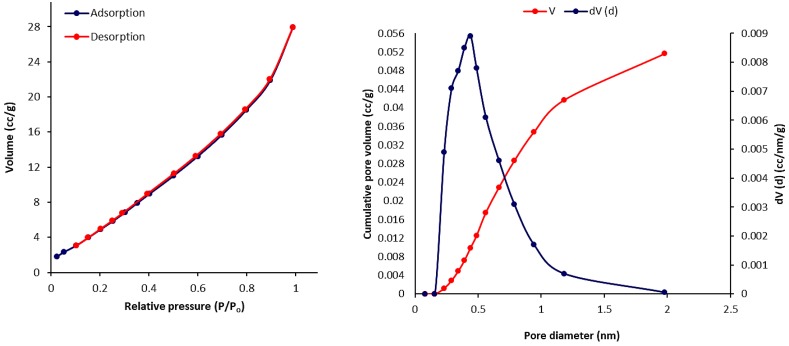
Adsorption–desorption isotherms of N_2_ and pore size distribution curve for **5**.

**Figure 5:**
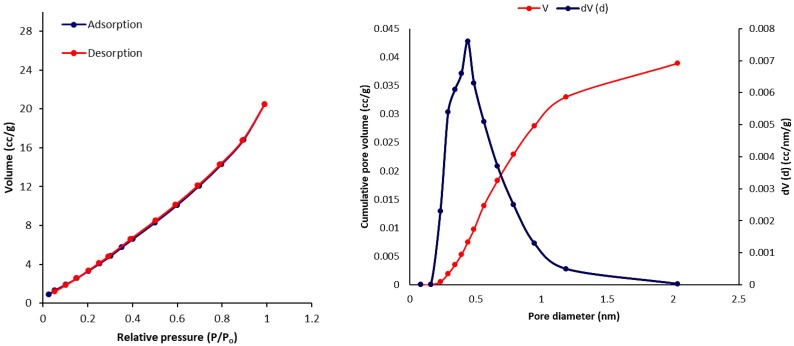
Adsorption–desorption isotherms of N_2_ and pore size distribution curve for **6**.

The N_2_ adsorption–desorption measurements were studied at 77 K to investigate the pore textural properties of polymers **4**–**6**. The adsorption desorption isotherms of N_2_ and pore size distribution curves for polymers **4**–**6** are shown in Figure 3, Figure 4 and Figure 5.

The synthesized polymer networks **4**–**6** show Type-III nitrogen sorption isotherms, which has no identifiable monolayer formation. The adsorbent–adsorbate interactions also appear to be relatively weak and the adsorbed molecules are clustered around the most favorable sites on the surface [54] indicating that the polymers have mesoporous structures. The apparent surface area of **4**–**6**, calculated from the Brunauer–Emmett–Teller (S_BET_), were 27.514, 30.021 and 24.840 m^2^·g^–1^, respectively. Polymers **4**–**6** have pore volumes 0.036, 0.052 and 0.040 cm^3^·g^–1^, respectively (Table 5).

**Figure 6:**
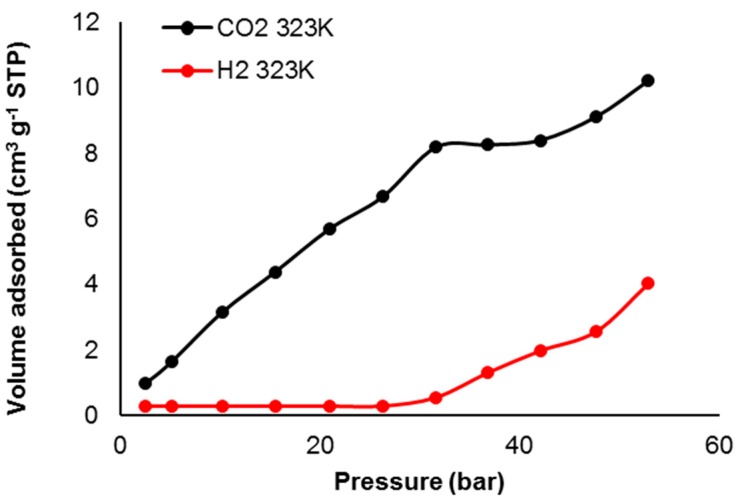
Adsorption isotherms of CO_2_ and H_2_ for polymer **4**.

**Figure 7:**
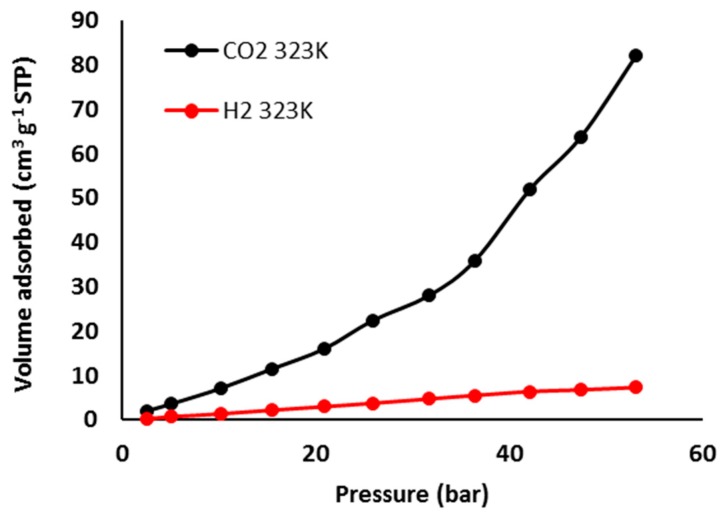
Adsorption isotherms of CO_2_ and H_2_ for polymer **5**.

**Figure 8:**
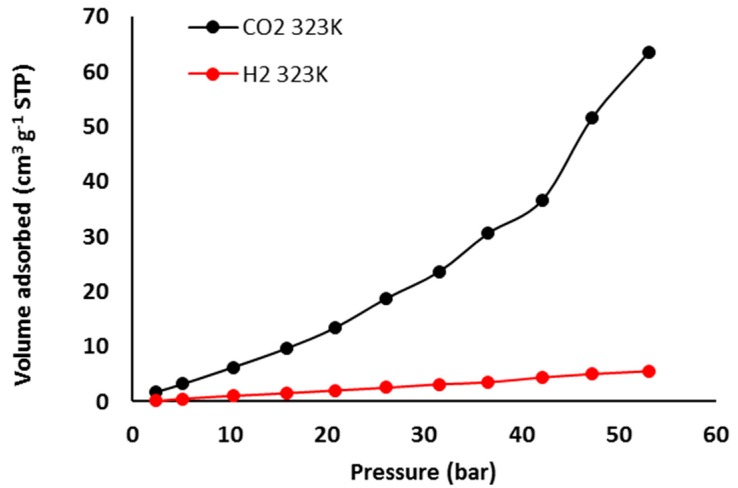
Adsorption isotherms of CO_2_ and H_2_ for polymer **6**.

The sorption of each polymer was studied at 323 K under 50 bars. The CO_2_ and H_2_ sorption isotherms of polymers **4**–**6** are shown in Figure 6, Figure 7 and Figure 8 and the data were recorded in Table 6. There is no apparent adsorption–desorption hysteresis, indicating that CO_2_ and H_2_ can be reversibly adsorbed in the pores under the chosen temperature and pressure. Polymers **4**–**6** show a high CO_2_ uptake. The CO_2_ adsorption quantity was 10.2, 82.1 and 63.4 cm^3^·g^–1^ for **4**, **5** and **6**, respectively (Table 5). Polymer **5** exhibits the highest BET surface area, total pore volume, H_2_ storage and CO_2_ uptake among tested polymers. Under the same condition, the polymers show low adsorption for H_2_ as 4.0, 7.4 and 5.5 cm^3^·g^–1^, respectively. Such behavior is due to the unfavorable interactions between hydrogen and the polymers.

Three monomers with different geometrical configurations were selected as the building block to produce polymers **4**–**6**, which have tetrahedral shape with sp^3^-hybridized phosphorus core. Linking the monomers with different geometries may tune the porosity and functionality of the synthesized polymeric materials [55]. Tetrahedral monomers are of interest since the porous polymers can be designed to possess high specific surface area that lack the flexibility to pack efficiently and leads to the facile formation of free volumes to promote the porosity [22,34,55,56,57,58,59,60,61]. Three-dimensional polymers possess stronger sorption ability for CO_2_ compared to H_2_. The heteroatoms (oxygen, sulfur or nitrogen) in POPs were reported to play an important role in the efficient capture of polar gases and adsorption selectivity of CO_2_ over N_2_ or CH_4_ [62,63,64,65]. The CO_2_ uptake for the most common MOF materials is up to 18 wt % at different temperature and pressure [37,66]. However, the health and environmental impacts of such materials are questionable [67].

The efficiency of the synthesized polymers was found to follow the order **5** > **6** > **4**. The difference in gas capture efficiency could be explained by the structure geometry of the building units for **4**–**6**. The kinked geometry of **5** (*meta*-phosphate) and **6** (*ortho*-phosphate) could lead to a distorted networks compared to polymer **4** (*para*-phosphate) that has a relatively less distorted structure. The synthesized polymers are suitable for CO_2_ capture as a result of the high number of Lewis base sites. In addition, the interaction between polarized groups and gas molecules increases the CO_2_ uptake of 14 wt % at 50 bars. Moreover, the van der Waals interaction between the skeleton and weak nonpolar CO_2_ might be stronger compared to those between the skeleton and strong nonpolar H_2_.

## 4. Conclusions

Three novel 3D polymeric Schiff bases containing phosphate group were successfully synthesized in high yields. The chemical structures of the synthesized polymers were confirmed by FTIR, SEM, GPC, BET, BJH and H-sorb2600 analyzer. The analysis of N_2_ sorption isotherms reveal that the three polymers have type III isotherm and quite narrow pore size distribution. The pronounced affinity towards CO_2_ gas uptake was found to be high compared to that of H_2_ uptake at 323 K and 50 bars. The outstanding storage of CO_2_ gas endows these polymers a promising potential as efficient adsorbents in clean energy applications.

## Figures and Tables

**Table 1:** Some FT-IR spectral data and elemental analyses for phosphate esters **1**–**3**.

Ester	FR-IR (*υ*, cm^−1^)				Elemental Analyses (%) Found (Calcd)	
	P–O–C	P=O	C=C	C=O	C	H
**1**	1161	1219	1600	1681	61.64 (61.47)	3.74 (3.68)
**2**	1188	1276	1585	1693	61.57 (61.47)	3.81 (3.68)
**3**	1172	1284	1581	1687	61.61 (61.47)	3.85 (3.68)

**Table 2:** ^1^H NMR spectral data for phosphate esters **1**–**3**.

Ester	^1^H NMR (400 MHz: DMSO-*d*_6_, δ, ppm, *J* in Hz)
**1**	9.79 (s, 3 H, CHO), 7.74 (d, *J* = 8.5 Hz, 6 H, Ar), 6.93 (d, *J* = 8.5 Hz, 6 H, Ar)
**2**	9.91 (s, 3 H, CHO), 7.41 (t, *J* = 8.2 Hz, 3 H, Ar), 7.36 (d, *J* = 8.2 Hz, 3 H, Ar), 7.24 (s, 3 H, Ar), 7.08 (d, *J* = 8.2 Hz, 3 H, Ar)
**3**	10.30 (s, 3 H, CHO), 7.71 (d, *J* = 8.3 Hz, 3 H, Ar), 7.58 (t, *J* = 8.3 Hz, 3 H, Ar), 7.05 (d, *J* = 8.3 Hz, 3 H, Ar), 7.02 (t, *J* = 8.3 Hz, 3 H, Ar)

**Table 3:** Some FT-IR spectral data for phosphate polymers **4**–**6**.

Polymer	FR-IR (*υ*, cm^−1^)			
	P–O–C	P=O	C=C	CH=N
**4**	1219	1172	1600	1635
**5**	1219	1087	1604	1635
**6**	1280	1172	1570	1616

**Table 4:** Qualitative molecular weight and polydispersity of phosphate polymers **4**–**6**.

Polymer	*Mw*	*Mn*	*Dp*	*Rt* (min)
**4**	12,700	12,000	1.05	17.20
**5**	12,400	11,100	1.11	17.19
**6**	26,800	25,900	1.03	16.61

**Table 5:** Surface area and porosity parameters of **4**–**6** obtained by the N_2_ adsorption.

Polymers	*S*_BET_ (m^2^·g^–1^) ^a^	*V*_total_ (cm^3^·g^–1^) ^b^	Pore Size (nm) ^c^
**4**	27.514	0.036	2.851
**5**	30.021	0.052	2.435
**6**	24.840	0.040	2.856

^a^ BET surface area; ^b^ Pore volume calculated from nitrogen adsorption at *P/P*_o_ = 0.9; ^c^ BJH average pore diameter calculated from desorption data.

**Table 6:** Gas uptake values for the polymer networks **4**–**6** ^a^.

Polymer	H_2_ Uptake (cm^3^·g^–1^) ^b^	H_2_ Uptake (wt %)	CO_2_ Uptake (cm^3^·g^–1^)	CO_2_ Uptake (wt %) ^c^
**4**	4.0	<0.050	10.2	1.8
**5**	7.4	0.066	82.1	14
**6**	5.5	0.050	63.4	11

^a^ The data were collected at 323 K and 50 bar; ^b^ The data were collected by volumetric H_2_ sorption method; ^c^ The data were collected by volumetric CO_2_ sorption method.
